# Renal Involvement and HBV Infection Are Common in Chinese Patients With Cryoglobulinemia

**DOI:** 10.3389/fimmu.2021.580271

**Published:** 2021-02-25

**Authors:** Wei Bai, Lixia Zhang, Jiuliang Zhao, Shangzhu Zhang, Jiaxin Zhou, Xiaomei Leng, Zhengyin Liu, Wenling Ye, Bing Han, Xinping Tian, Mengtao Li, Yan Zhao, Xiaofeng Zeng

**Affiliations:** ^1^Department of Rheumatology, Peking Union Medical College Hospital, Peking Union Medical College & Chinese Academy of Medical Science, Beijing, China; ^2^Key Laboratory of Rheumatology and Clinical Immunology, Ministry of Education, National Clinical Research Center on Rheumatology, Ministry of Science and Technology, Beijing, China; ^3^Department of Rheumatology, Shunyi District Hospital, Beijing, China; ^4^Department of Infectious Disease, Peking Union Medical College Hospital, Peking Union Medical College & Chinese Academy of Medical Science, Beijing, China; ^5^Department of Nephrology, Peking Union Medical College Hospital, Peking Union Medical College & Chinese Academy of Medical Science, Beijing, China; ^6^Department of Haematology, Peking Union Medical College Hospital, Peking Union Medical College & Chinese Academy of Medical Science, Beijing, China

**Keywords:** cryoglobulinemia, vasculitis, HBV infection, autoimmunity diseases, renal involvement

## Abstract

**Objectives:** This study aimed to describe the main characteristics of Chinese patients with cryoglobulinemia, especially the characteristics of patients with different causes of cryoglobulinemia.

**Methods:** Eighty inpatients diagnosed with cryoglobulinemia from different wards in Peking Union Medical College Hospital were included in this study. Demographic, clinical, biological, and renal pathological data were collected. We analyzed the characteristics of 61 patients with different causes of cryoglobulinemia.

**Results:** Most patients (36/80, 45%) were diagnosed between 40 and 60 years of age. The male: female ratio was 1:1.5. Mixed (II + III) cryoglobulinemia accounted for the majority (43.8%) of cases. Renal involvement (87.5%), cutaneous involvement (57.5%), and fever (27.5%) were the most common clinical manifestations, while other manifestations included serositis and pulmonary and gastrointestinal involvement. The most common renal histopathological pattern was membranoproliferative glomerulonephritis (25/42, 59.5%). The secondary causes of cryoglobulinemia included infectious diseases (26/61, 32.5%), such as hepatitis B virus (HBV) and hepatitis C virus (HCV) infections, and connective tissue diseases (22/61, 27.5%), such as lupus and hematologic tumors (13/61, 16.3%). Patients with hematologic tumors were diagnosed at an older age (*P* = 0.044) and mostly had type I cryoglobulinemia (*P* < 0.001). No significant difference in clinical or biological manifestations was found among patients with different causes of cryoglobulinemia.

**Conclusions:** This is the largest cohort of Chinese patients with cryoglobulinemia. We found that renal involvement and HBV infection might be more common in Chinese patients with cryoglobulinemia.

## Introduction

Cryoglobulins are circulating immunoglobulins that precipitate at low temperatures and dissolve with rewarming. We generally use Brouet's classification to define various types of cryoglobulinemia (1). Type I cryoglobulinemia is characterized by a single monoclonal immunoglobulin, commonly IgM or IgG. Type II cryoglobulinemia and III cryoglobulinemia are referred to as mixed cryoglobulinemia. Type II cryoglobulins include immune complexes comprising oligoclonal IgM and polyclonal immunoglobulins (mainly IgG), and type III cryoglobulins include polyclonal immunoglobulins (generally IgM). In most patients with this condition, there are secondary causes of cryoglobulinemia. Type I cryoglobulinemia is often associated with hematological malignancies, while types II and III are associated with infectious diseases and autoimmune diseases (2). In addition, some patients present with idiopathic or essential cryoglobulinemia.

According to the 2012 Chapel Hill definition of vasculitis, cryoglobulinemia is one of the common causes of immune complex small-vessel vasculitis (3). In patients with cryoglobulinemic vasculitis, cryoglobulin immune deposits are always seen in small vessels. Cutaneous, articular, and renal involvement and neuropathy are the most common clinical manifestations (4). There have been many studies on cryoglobulinemia patients. In 1995, the first large cohort included 913 Italian cryoglobulinemia patients; that study compared the clinical and laboratory data reported by the Italian Group for the Study of Cryoglobulinemias (GISC) (5). Since then, an increasing number of studies from European countries have been published. In the CryoVas survey from France, 342 mixed cryoglobulinemia patients were evaluated, of which 18 patients had non-HCV (hepatitis C virus) infectious diseases and 242 patients had non-infectious diseases (6, 7). A Spanish research study analyzed the clinical manifestations and prognosis of 279 HCV infection patients presenting with life-threatening cryoglobulinemic vasculitis (8). Another single-center cohort in Italy enrolled 246 patients with mixed cryoglobulinemia and analyzed their clinical manifestations, survival, and prognostic factors (9).

As mentioned above, many patients with infectious diseases or connective tissue diseases may have cryoglobulinemia. They may show a poor prognosis if organs such as the kidneys or nervous system are involved. Many studies worldwide have analyzed the characteristics of patients with cryoglobulinemia. However, data on Chinese patients are limited. Hence, this retrospective study aimed to analyze the characteristics of cryoglobulinemia in Chinese patients, especially patients with the involvement of important organs or with various secondary causes, and to compare our results with those of other studies from different countries. Our research may provide a better understanding of this disease.

## Materials and Methods

### Patient Recruitment and Follow-Up

We reviewed all inpatient medical records from January 2001 to August 2018 in Peking Union Medical College Hospital (PUMCH). The inclusion criteria for the study were as follows: (1) cryoglobulin positivity (cryocrit > 1.0%) and a clinical diagnosis of cryoglobulinemia; (2) aged ≥18 years (male or female); and (3) admission to the ward and complete inpatient medical records. The exclusion criteria was no inpatient medical records but only outpatient clinic medical records. Most patients came from the internal medicine, hematology, nephrology, and rheumatology wards of our hospital. Demographic data included data on sex and the age at diagnosis. If the patient wasn't regularly followed up in the outpatient clinic after discharge from ward, we conducted a follow-up interview via telephone during March 2019 to July 2019. We asked the patients or their relatives questions regarding the patient's health since the last hospital visit. Main questions included survival, medication and chief complaint of the patient. We summarized and analyzed other clinical and biological information, mainly extracted from the medical records in PUMCH. This study was approved by the Medical Ethics Committee of PUMCH (approval number: S-K992).

### Clinical Assessment

Patients with no other relevant diseases were classified as having idiopathic or essential cryoglobulinemia. Others were classified to have cryoglobulinemia due to secondary causes, which included infectious diseases, connective tissue diseases, and hematologic diseases. We also found that some patients had two or more possible causes of cryoglobulinemia. In this scenario, we discussed the case with two or more experienced clinicians from different departments based on the patients' clinical and biological manifestations and identified the dominant cause.

The following clinical data were collected and compared among groups with different causes of cryoglobulinemia: fever, cutaneous involvement, arthralgia, renal involvement (proteinuria, microscopic hematuria, elevated serum creatinine levels), neurologic involvement (mainly peripheral neuropathy), gastrointestinal involvement (gastrointestinal ulcer or bleeding), serositis, and pulmonary involvement (pulmonary hypertension, interstitial lung disease, and pulmonary alveolar hemorrhage).

### Laboratory Parameters

We used modified capillary immunotyping (SEBIA Company, Paris, France) or immunofixation electrophoresis for cryoglobulin identification. Cryoglobulin positivity was defined as a cryocrit level >1.0% in serum samples. Other biological features included cryoglobulin type, anemia, thrombocytopenia, leukopenia, hypocomplementemia, hyperglobulinemia, and rheumatoid factor positivity. Serum levels of the complement components C3 and C4 and immunoglobulins including IgA, IgG, and IgM were evaluated and compared among patients with different causes of cryoglobulinemia. Data regarding histopathological patterns observed in renal biopsy specimens were collected from patients showing renal involvement who underwent renal puncture biopsy.

### Statistical Analysis

Continuous variables were presented as the mean ± standard deviation if they were normally distributed or as the median and interquartile range if they had a skewed distribution. Categorical variables were presented as absolute and relative frequencies. One-way analysis of variance test or the rank sum test was used to compare continuous variables, where appropriate, and an χ^2^ test was used to compare categorical variables. All *P*-values were two-tailed, and the level of significance was set at 0.05. All calculations were performed using the SPSS 23.0 statistical package (SPSS, Chicago, IL, USA).

## Results

### Main Characteristics of Patients With Cryoglobulinemia

Altogether, 80 patients treated in PUMCH from January 2001 to August 2018 were included. Most patients (36/80, 45%) were diagnosed between 40 and 60 years of age (Table 1). The male: female ratio was 1:1.5. Moreover, 47 (58.7%) patients had specific types of cryoglobulinemia: 12 (15.0%) had type I cryoglobulinemia, 15 (18.8%) had type II cryoglobulinemia, and 20 (25%) had type III cryoglobulinemia. In total, 19 (23.8%) patients had idiopathic (essential) cryoglobulinemia; the other 61 (76.3%) patients had cryoglobulinemia due to secondary causes.

**Table 1:** Main characteristics of 80 patients with cryoglobulinemia.

	***n* (%)**
**Age at diagnosis, year**	
<20	4 (5.0%)
20–40	15 (18.8%)
40–60	36 (45.0%)
>60	25 (31.3%)
Female gender	48 (60.0%)
**Type of cryoglobulins**	
I	12 (15.0%)
II	15 (18.8%)
III	20 (25.0%)
Possible but no specific type	33 (41.3%)
Secondary causes	61 (76.3%)
Infectious disease	26 (32.5%)
HCV	15
HBV	10
HCV and HBV	1
Connective tissue disease	22 (27.5%)
SLE	13
Hematologic disease	13 (16.3%)
**Outcome**	
Survive	61 (76.3%)
Death	5 (6.3%)
Loss of follow up	14 (17.5%)

### Clinical, Laboratory, and Pathological Features in Patients With Cryoglobulinemia

Renal involvement (70/80, 87.5%), cutaneous involvement (46/80, 57.5%), and fever (22/80, 27.5%) were the most common clinical manifestations, followed by arthralgia (20/80, 25.0%), nervous system involvement (14/80, 17.5%), serositis (16/80, 20.0%), pulmonary involvement (9/80, 11.3%), and gastrointestinal involvement (6/80, 7.5%) (Table 2).

**Table 2:** Clinical and laboratory features of 80 patients with cryoglobulinemia.

	***n* (%)**
**Clinical manifestations**	
Fever	22 (27.5%)
Cutaneous	46 (57.5%)
Arthralgia	20 (25.0%)
Kidney	70 (87.5%)
Microscopic hematuria	54 (67.5%)
Proteinuria (24 h UP > 0.5 g)	60 (75.0%)
Elevated serum creatinine	43 (53.8%)
Nervous system	14 (17.5%)
Peripheral neuropathy	11 (13.8%)
Pulmonary	9 (11.3%)
Pulmonary hypertension	5 (6.3%)
Interstitial lung disease	3 (3.8%)
Pulmonary alveolar hemorrhage	1 (1.3%)
Gastrointestinal (GI ulcer or bleeding)	6 (7.5%)
Serositis	16 (20.0%)
**Laboratory parameters**	
Abnormal complete blood count	71 (88.8%)
Anemia	64 (80.0%)
Leukopenia	16 (20.0%)
Thrombocytopenia	21 (26.3%)
**Hypocomplementemia**	
C4	50 (62.5%)
C3	52 (65.0%)
Hyperglobulinemia	
IgM	37 (46.3%)
IgG	20 (25.0%)
IgA	9 (11.3%)
Rheumatoid factor positive	48 (60.0%)

In the cohort, 71 (88.8%) patients had an abnormal complete blood count: 64 (80%) patients had anemia, 21 (26.3%) had thrombocytopenia, and 16 (20%) had leukopenia. Additionally, 50 (62.5%) patients had decreased C4 complement levels, while 52 (65%) had decreased C3 complement levels; 37 (46.25%) patients had increased IgM levels, while 20 (25%) had increased IgG levels and 9 (11.25%) had increased IgA levels; and 48 (60%) patients had rheumatoid factor positivity.

Furthermore, 42 patients underwent renal puncture biopsy. The most common renal histopathological pattern was membranoproliferative glomerulonephritis (MPGN) (25/42, 59.5%), followed by membranous nephropathy (MN) (6/42, 14.3%), lupus nephritis (LN) (5/42, 11.9%), and rapidly progressive glomerulonephritis (RPGN) (2/42, 4.7%). The other histopathological patterns (4/42, 9.5%) included focal segmental glomerulosclerosis (FSGS), acute tubular necrosis, plasma cell disease-related renal impairment, and minimal change nephropathy.

### Patients With Different Causes of Cryoglobulinemia

Of the 80 patients, 61 (76.3%) had secondary causes of cryoglobulinemia, which could be divided into three groups: infectious diseases (26/61, 32.5%), connective tissue diseases (22/61, 27.5%), and hematologic malignancies (13/61, 16.3%). Infectious diseases included hepatitis B virus (HBV) infection (10/26), HCV infection (15/26), and co-infection with HCV and HBV (1/26). Connective tissue diseases included systemic lupus erythematosus (13/22), primary Sjogren's syndrome (3/22), rheumatoid arthritis (2/22), and others (4/22). Hematologic malignancies included lymphoma (11/13), myeloma (1/13), and leukemia (1/13).

Patients with hematological malignancies were diagnosed at an older age than those with infectious and connective tissue diseases (*P* = 0.044, Table 3). There was no difference in the sex ratio among the three groups. The rate of type I cryoglobulinemia was higher in patients with hematologic tumors (*P* < 0.001), while the rate of mixed (type II + III) cryoglobulinemia was higher in patients with connective tissue diseases and infectious diseases (*P* = 0.012). There was no significant difference in clinical manifestations among the three groups. The rate of thrombocytopenia was higher in patients with hematological malignancies, and serum IgG and IgM levels were higher in patients with connective tissue diseases and hematologic malignancies; however, the differences were not significant (rate of thrombocytopenia, *P* = 0.058; serum IgG, *P* = 0.166, and serum IgM, *P* = 0.071).

**Table 3:** Clinical and laboratory characteristics of 61 patients with different causes of cryoglobulinemia.

	**Infectious disease**	**Connective tissue disease**	**Hematologic tumor**	***P*-value**
Numbers	26	22	13	
Age at diagnosis, year	49.9 ± 14.8	43.9 ± 18.2	58.2 ± 13.6	0.044
Female gender	18 (69.2%)	15 (68.2%)	6 (46.2%)	0.321
**Type of cryoglobulins**				
I	1 (3.8%)	0 (0.0%)	6 (46.2%)	<0.001
II+III	9 (34.6%)	16 (72.7%)	4 (30.8%)	0.012
**Clinical manifestations**				
Fever	6 (23.0%)	7 (31.8%)	3 (23.1%)	0.758
Cutaneous	16 (61.5%)	11 (50.0%)	8 (61.5%)	0.682
Arthralgia	8 (30.8%)	7 (31.8%)	2 (15.4%)	0.525
Renal involvement	24 (92.3%)	19 (86.4%)	9 (69.2%)	0.157
Proteinuria(24 h UP > 0.5 g)	21 (80.8%)	15 (68.2%)	8 (61.5%)	0.211
Elevated serum creatinine	12 (46.7%)	12 (54.5%)	7 (53.8%)	0.820
Nervous system	4 (15.4%)	4 (18.2%)	4 (30.8%)	0.510
Serositis	6 (23.1%)	6 (27.3%)	3 (23.1%)	0.935
**Laboratory parameters**				
Anemia	20 (76.9%)	18 (81.8%)	12 (92.3%)	0.499
Thrombocytopenia	6 (23.1%)	4 (18.2%)	7 (53.8%)	0.058
C3 complement (g/L)	0.70 ± 0.35	0.53 ± 0.34	0.55 ± 0.22	0.174
C4complement (g/L)	0.11 ± 0.12	0.06 ± 0.06	0.08 ± 0.07	0.529
IgG level (g/L)	10.1 ± 6.3	15.8 ± 10.5	12.6 + 8.3	0.166
IgM level (g/L)	4.0 ± 5.4	2.3 ± 1.8	4.0 ± 6.1	0.371
IgA level (g/L)	1.6 ± 1.16	2.5 ± 1.4	2.5 ± 1.6	0.071

### Outcomes

Of the 80 patients, 61 (76.3%) survived, and 14 (17.5%) were lost to follow-up. Five patients (6.3%) died (Table 4).

**Table 4:** Main characteristics of the five dead patients.

**Patient**	**Gender**	**Type of cryoglobulinemia**	**Secondary cause of cryoglobulinemia**	**Age at diagnose**	**Age of death**	**Death cause**
1	Male	II	–	69	70	Septic shock
2	Female	III	Connective tissue disease	19	19	Septic shock
3	Male	Unknown type	Connective tissue disease	61	61	Septic shock
4	Female	Unknown type	Hepatocellular carcinoma	77	81	Hepatocellular carcinoma
5	Female	Unknown type	HBV infection	53	55	Unknown reason

## Discussion

To our knowledge, this study includes the largest cohort of Chinese patients with cryoglobulinemia to date. The sex ratio was nearly the same as that reported previously (10). Our patients were diagnosed at a younger age than those in other cohorts (6, 9). This might be because the majority of our patients were diagnosed with infectious and connective tissue diseases, and these patients are generally younger than patients with hematological tumors. Mixed (II + III) cryoglobulinemia accounted for the majority (43.8%) of cases in our cohort. However, due to laboratory limitations, only serum cryoglobulin positivity without specific classification was detected in many patients, especially those diagnosed in earlier years. This had a certain impact on data analysis. Patients with hematologic tumors had a significantly higher rate of type I cryoglobulinemia, while the others had higher rates of type II and type III cryoglobulinemia. This result and the biological characteristics of cryoglobulinemia were consistent with those from other reports (10).

Renal involvement (87.5%) was the most common clinical manifestation of cryoglobulinemia in our cohort. The prevalence of renal involvement in our cohort was higher than that in other studies (14–63%) (11, 12). Since we only included patients who were admitted to the ward, there may have been a selection bias toward severe patients with involvement of an important organ, such as the kidneys. This may be the reason for the high prevalence of renal involvement. A retrospective study of 153 French patients, in which 45 patients had renal involvement, showed that type II cryoglobulinemia, a high serum cryoglobulin concentration, the presence of an IgG kappa monoclonal component, and diabetes were independently associated with the risk of renal involvement, and no particular etiology independently predicted renal involvement (13). In our cohort, we found no differences in clinical manifestations among the three groups of patients based on secondary causes. There may be a trend that patients with infectious and connective tissue diseases showed renal involvement more commonly than patients with hematological malignancies (92.3 and 86.4% vs. 69.2%), although the difference was not significant.

Of the 70 patients with renal involvement, 42 (60%) had available results for the pathological examination of renal biopsy specimens. Previous studies have shown that the most common renal pathology pattern in patients with cryoglobulinemia was MPGN, and other studies have reported mesangial proliferative glomerulonephritis (MsPGN), MN, FSGS, fibrillary or immunotactoid glomerulopathy, and thrombotic microangiopathy (TMA) (14, 15). In a French cohort, 14 patients underwent renal biopsy, the majority of whom had MPGN (13/14) (13). Here in our study, MPGN was the most common renal pathology pattern, followed by lupus nephritis, MN, and RPGN. This result might be related to the various underlying diseases in this cohort, such as lupus and infectious diseases. Meanwhile, the pathological types of glomerulonephritis caused by cryoglobulinemia might have varied.

Cutaneous involvement and articular and nervous system involvement are common manifestations in patients with cryoglobulinemia. In 2011, the classification criteria for cryoglobulinemia vasculitis included general symptoms (such as fever and fatigue), articular involvement, vascular involvement (such as purpura and skin ulcers), and nervous system involvement in the clinical items (16, 17). In previous reports, the prevalence of articular involvement was reported to be 40–75%, and the prevalence of nervous system involvement was reported to be 17–60% (mainly peripheral neuropathy) (4, 6, 7, 12). Most studies have shown that the prevalence of cutaneous involvement was approximately 69%−90% (4, 7, 12, 18, 19), except for a Spanish single-center study, wherein only 24% of 443 patients had cutaneous involvement (20). It has been reported that purpura, cutaneous necrosis, and articular involvement are associated with early relapse (18), and in one study, significantly low survival rates were observed in patients with renal involvement (12).

In general, the prevalence rates of articular, nervous system, and cutaneous involvement in our cohort was lower than those in most other studies, while the prevalence of renal involvement was higher. As mentioned before, we only included patients who were admitted to the ward. This meant that only severe patients showing involvement of important organs were included. The prevalence of cutaneous or articular involvement or peripheral neuropathy may be higher in outpatients, and furthermore, those patients were not included in this cohort. This might be the reason for the differences. However, our results suggest that more attention needs to be paid to renal involvement in patients with cryoglobulinemia.

Rare clinical manifestations of cryoglobulinemia, such as pulmonary involvement, gastrointestinal involvement, and serositis, were found in our patients. Alveolar hemorrhage, organizing pneumonia, pulmonary vasculitis, and pleural effusion have been previously reported in patients with cryoglobulinemia (2, 21). In this study, we included patients with pulmonary hypertension, which has not been reported by previous studies. Among the five patients with pulmonary hypertension, one showed antiphospholipid antibody positivity and was suspected of having pulmonary vasculitis; one had HCV infection and moderate pulmonary hypertension due to tricuspid regurgitation; and the other three had mild pulmonary hypertension, as estimated by echocardiography. Thus, patients with cryoglobulinemia might show pulmonary vascular involvement due to underlying diseases, such as rheumatologic and infectious diseases. The prevalence of gastrointestinal involvement reported in another study was <5%, which was similar to our finding. Moreover, gastrointestinal involvement manifesting as gastrointestinal bleeding or perforation may be related to small vessel vasculitis (2, 4). Research on HCV-related systemic vasculitis has shown that patients with gastrointestinal manifestations have higher levels of mixed cryoglobulins (22). Furthermore, pulmonary and intestinal involvement might be life-threatening as many patients die from pulmonary hemorrhage and intestinal ischemia (21–23). There have been few reports on serositis in patients with cryoglobulinemia. However, as patients with cryoglobulinemia may have hepatitis, connective tissue disease, or tumors, serositis associated with these diseases might not be uncommon.

According to previous reports and our results, an elevated immunoglobulin level, especially an elevated IgM level, hypocomplementemia, and rheumatoid factor positivity were common laboratory manifestations of cryoglobulinemia (21). Interestingly, the rates of reduced C3 and C4 levels were similar in our cohort, whereas C4 reduction was more prominent in other studies (20). This may be related to the higher proportion of patients with connective tissue diseases in our study. The serum immunoglobulin levels of patients with cryoglobulinemia have rarely been mentioned in previous reports. In our cohort, patients with infectious diseases had lower IgG and IgA levels than patients with connective tissue diseases and hematologic tumors, although this difference was not significant. This might be due to the reduction in immunoglobulin synthesis and secretion in patients with hepatitis.

Infectious diseases, mainly HCV infection, have been reported as the most common causes of cryoglobulinemia in previous studies, followed by autoimmune diseases and hematological tumors. In two large cohorts involving 443 and 1,434 patients, the proportions of patients with infectious diseases were 75 and 92%, respectively, while those of patients with HCV infection were 73 and 91%, respectively (20, 24, 25). Data on HBV-related cryoglobulinemia are limited. An epidemiologic study from the USA published in 1990 involved 1,400 HBV infection patients and found no cases of mixed cryoglobulinemia (26). A data analysis of the GISC cohort published in 1992 did not support an association between HBV infection and cryoglobulinemia (27). Studies in Italy and Spain have shown that patients with HBV infection accounted for only 3% of patients with cryoglobulinemia (9, 20). However, there have been some reports focusing on HBV-related cryoglobulinemia. In 2016, another GISC-based study summarized 17 cases of HBV-related cryoglobulinemia (28). China is the most populous country worldwide and has a considerable number of patients with HBV infection. Data in 2017 showed that the prevalence of HBsAg positivity in the general Chinese population was 7.18% (29). During these years, studies from different centers in China have focused on HBV-associated cryoglobulinemia, especially in patients with renal involvement (30, 31). Some studies have reported that the prevalence of HBV infection in cryoglobulinemia patients may be equal to or greater than that of HCV infection in cryoglobulinemia patients in China (32–34). This study found similar results. HBV-related cryoglobulinemia may not be uncommon, especially in China.

According to previous studies, Sjogren's syndrome was the most common autoimmune disease causing cryoglobulinemia, followed by lupus (19, 20). In our cohort, lupus was the most common autoimmune disease causing cryoglobulinemia. One study showed that cryoglobulins were detected in 25% of 122 lupus patients (35), suggesting that cryoglobulinemia might be common in lupus patients.

Meanwhile, some patients with secondary causes had two or more causative diseases. Three patients had hematologic tumors and HBV infection, one had a hematologic tumor and HCV infection, five had connective tissue diseases and HBV/HCV infection, and two had connective tissue diseases and hematologic tumors. After discussion with experienced doctors from relevant departments, we identified the most likely cause of cryoglobulinemia in these patients. The clinical characteristics of patients with two or more causative diseases are complicated and require further discussion.

This study had some limitations. First, this was a single-center, retrospective study and only included patients admitted to the ward. Our hospital is a referral center for complicated and severe patients. Therefore, this study may have selection and referral biases, resulting in the inclusion of more severe patients with organ involvement, such as renal involvement, or the rare clinical manifestations of pulmonary and gastrointestinal involvement. Second, due to laboratory limitations, especially in the early years, cryoglobulinemia was not classified and the cryoglobulin concentration was not measured in some patients, which are important for determining the severity of cryoglobulinemia (13). Recently, we have started using the modified capillary immunotyping and immunofixation electrophoresis methods for identifying cryoglobulins and testing the absolute cryoglobulin concentration. However, the lack of cryoglobulin quantification in the early years might have had an impact on data analysis. Third, many patients were lost to follow-up. In a previous study, the 10 year survival rate of patients with type II cryoglobulinemia was 71%, and that of type III cryoglobulinemia was 84% (9). In our study, 6.3% of patients died, while 17.5% were lost to follow-up, some of whom may have died. Hence, a further detailed follow-up study of our patients may be helpful for analyzing 5 and 10 year survival rates and identifying prognostic factors in patients with cryoglobulinemia.

## Conclusions

We analyzed the demographic, clinical, and biological characteristics of patients with cryoglobulinemia in a single center in China. Some characteristics of our patients were different from those reported by previous studies. Renal involvement was common in cryoglobulinemia patients, and patients had various renal pathology patterns. Some rare clinical manifestations, such as pulmonary and gastrointestinal involvement, might be serious and life-threatening. Infectious and connective tissue diseases were the most common causes of cryoglobulinemia. Lupus was the most common autoimmune disease associated with cryoglobulinemia in our cohort. HBV infection was found to be an important cause of cryoglobulinemia in China.

## Data Availability Statement

The raw data supporting the conclusions of this article will be made available by the authors, without undue reservation.

## Ethics Statement

The studies involving human participants were reviewed and approved by PUMCH Institutional Review Board. Written informed consent for participation was not required for this study in accordance with the national legislation and the institutional requirements.

## Author Contributions

WB and LZ collected and analyzed the clinical data and did the follow-up interview. WB wrote and edited the manuscript. XL provided guidance and reviewed the manuscript. ZL, WY, and BH provided professional advices from other departments. JZhao, SZ, JZhou, XT, ML, YZ, and XZ contributed to study design and data analysis. All authors contributed to the article and approved the submitted version.

## Conflict of Interest

The authors declare that the research was conducted in the absence of any commercial or financial relationships that could be construed as a potential conflict of interest.
